# A Rare Case of Traumatic Bilateral Elbow Dislocation Without a Fracture in a Seven-Year-Old Female Child

**DOI:** 10.7759/cureus.19459

**Published:** 2021-11-11

**Authors:** Christos Topalis, Eustathios Kenanidis, Christos I Konstantinidis, Michael E Potoupnis, Eleftherios Tsiridis

**Affiliations:** 1 Academic Orthopaedic Department, Papageorgiou General Hospital, Aristotle University Medical School, Thessaloniki, GRC; 2 3rd Orthopaedic Department, Center of Orthopaedic and Regenerative Medicine, Center of Interdisciplinary Research and Innovation, Aristotle University Medical School, Thessaloniki, GRC

**Keywords:** closed reduction, fracture, pediatric, trauma, bilateral, elbow dislocation, child

## Abstract

Pediatric bilateral elbow dislocation is an infrequent injury. This is a report of a seven-year-old girl, the youngest patient ever reported, with simultaneous isolated bilateral traumatic elbow dislocation without fracture, treated with closed reduction under sedation.

## Introduction

Elbow trauma in children is one of the most commonly encountered musculoskeletal injuries in pediatric patients. Supracondylar fracture is the most common type of elbow fracture in children (60% of all pediatric elbow fractures) occurring in the ages of five to 10 years old with a typical mechanism of falling on an outstretched hand with extended elbow [1]. The exact mechanism is also the leading cause of elbow dislocations in children, which is a very infrequent injury, accounting for only 3%-6% of pediatric elbow injuries. Furthermore, bilateral elbow dislocation in pediatrics is an extremely rare injury, and reports of such injuries are very limited.

## Case presentation

A seven-year-old otherwise healthy female sustained bilateral elbow trauma after a fall with outstretched elbows and landing with force on the floor (kindergarten facility at the climbing frame).

The neurovascular status of both upper extremities was intact upon the arrival of the patient to the Trauma Unit. Clinical examination revealed loss of any active movement in both elbow joints in every plane. The joint was locked in a relatively extended position with the forearm neutral to a slightly supinated position. The patient had no sign of swelling or hematoma. Clinical suspicion was guided to a complex elbow injury, possibly with the participation of various bony structures.

A gross estimation of the patient's potential hyperlaxity was performed except for the elbow joints using the Beighton scale without significant clinical findings [2]. Neurovascular status of the upper limbs was thoroughly re-examined, but no sign of neural or vascular impairment or compromise was found.

**Figure 1:**
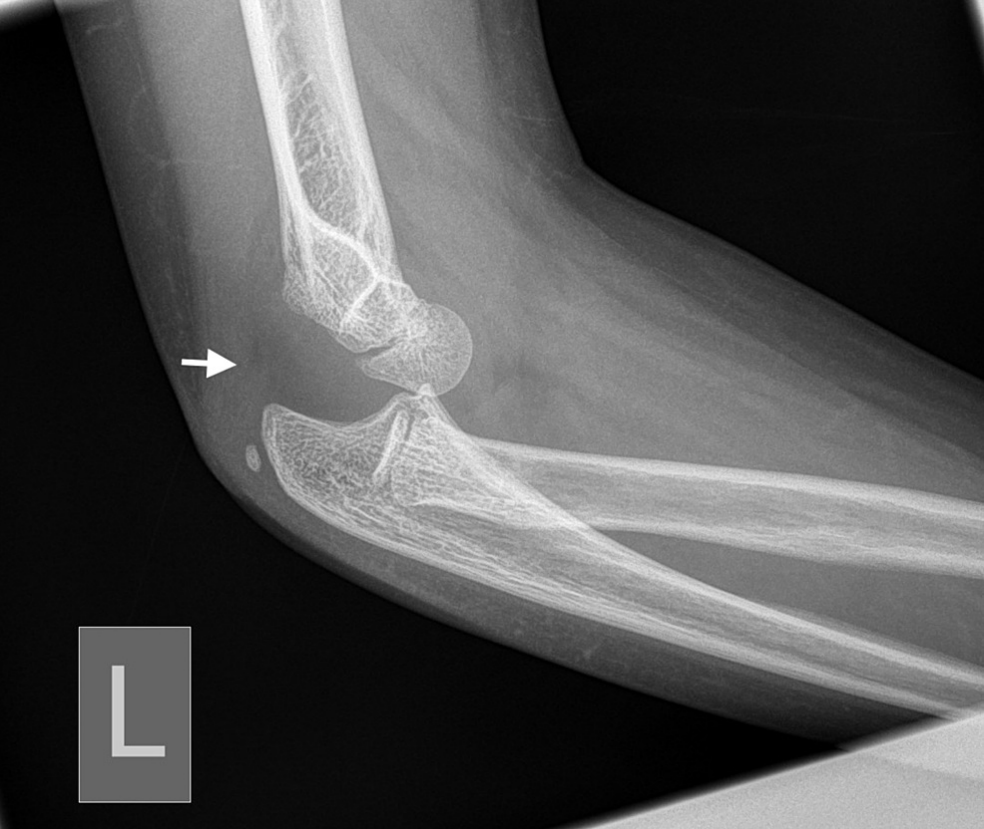
Left (L) posterior elbow dislocation (white arrow) without periarticular fracture (view 1).

**Figure 2:**
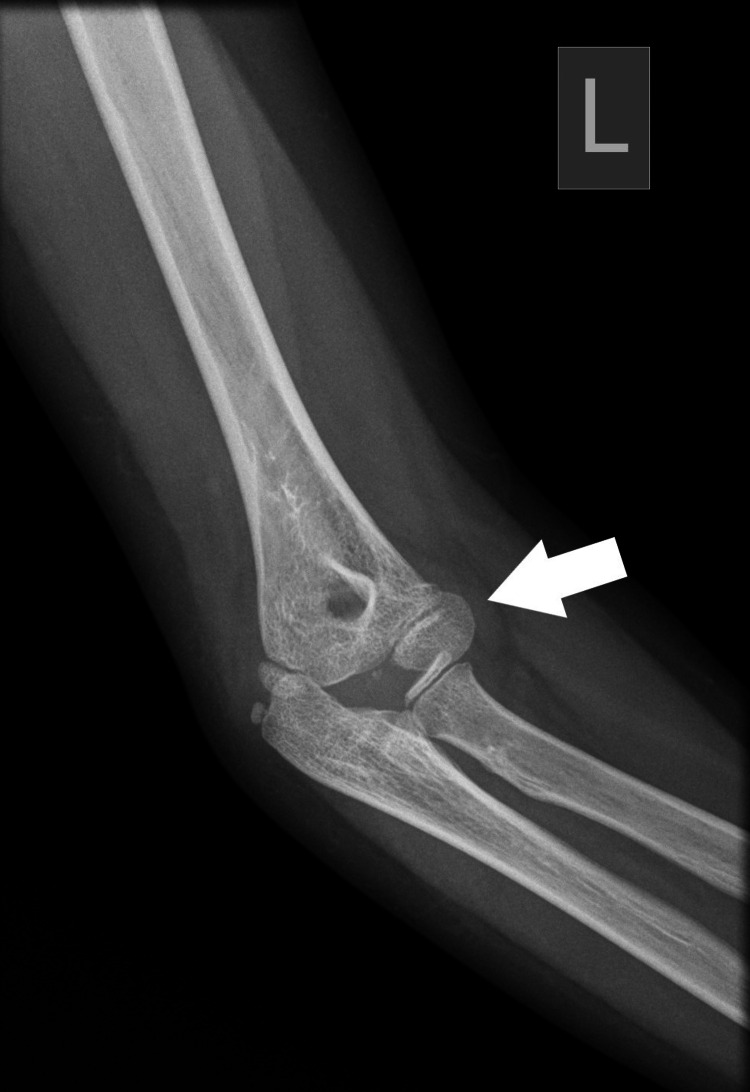
Left (L) posterior elbow dislocation (white arrow) without periarticular fracture (view 2).

**Figure 3:**
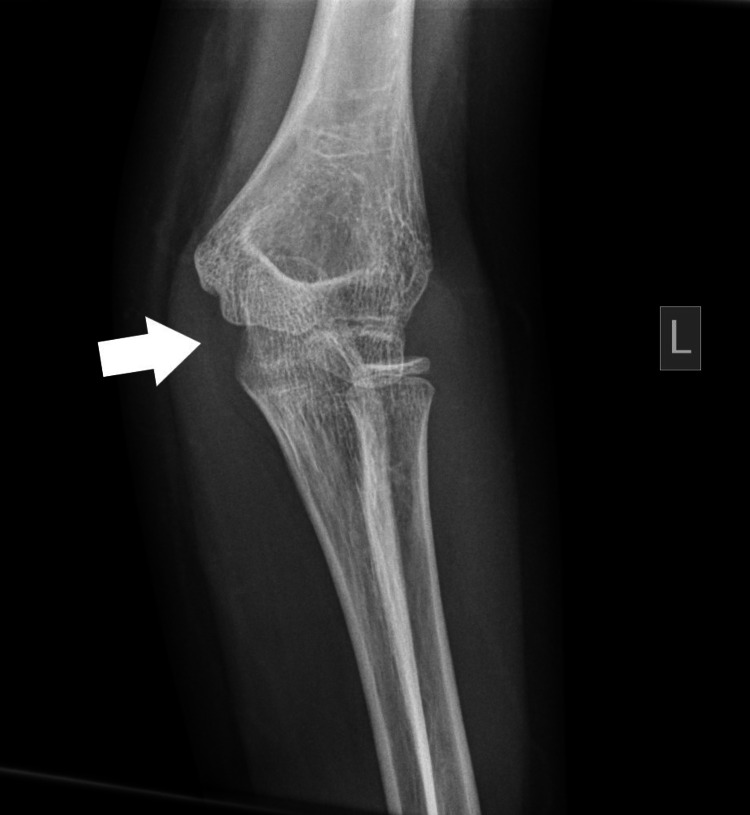
Left (L) posterior elbow dislocation (white arrow) without periarticular fracture (view 3).

**Figure 4:**
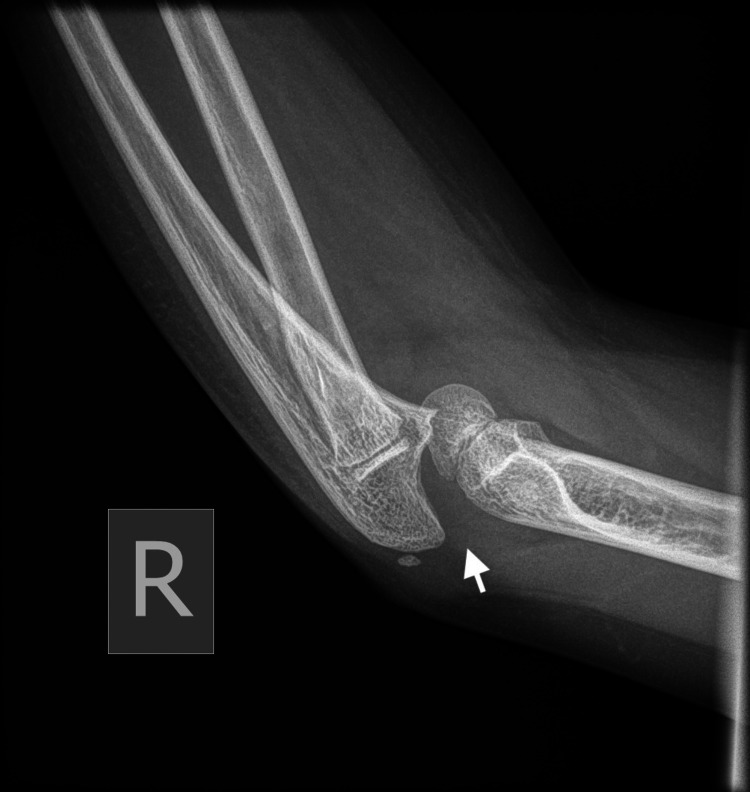
Right (R) posterior elbow dislocation (white arrow) without periarticular fracture (view 1).

**Figure 5:**
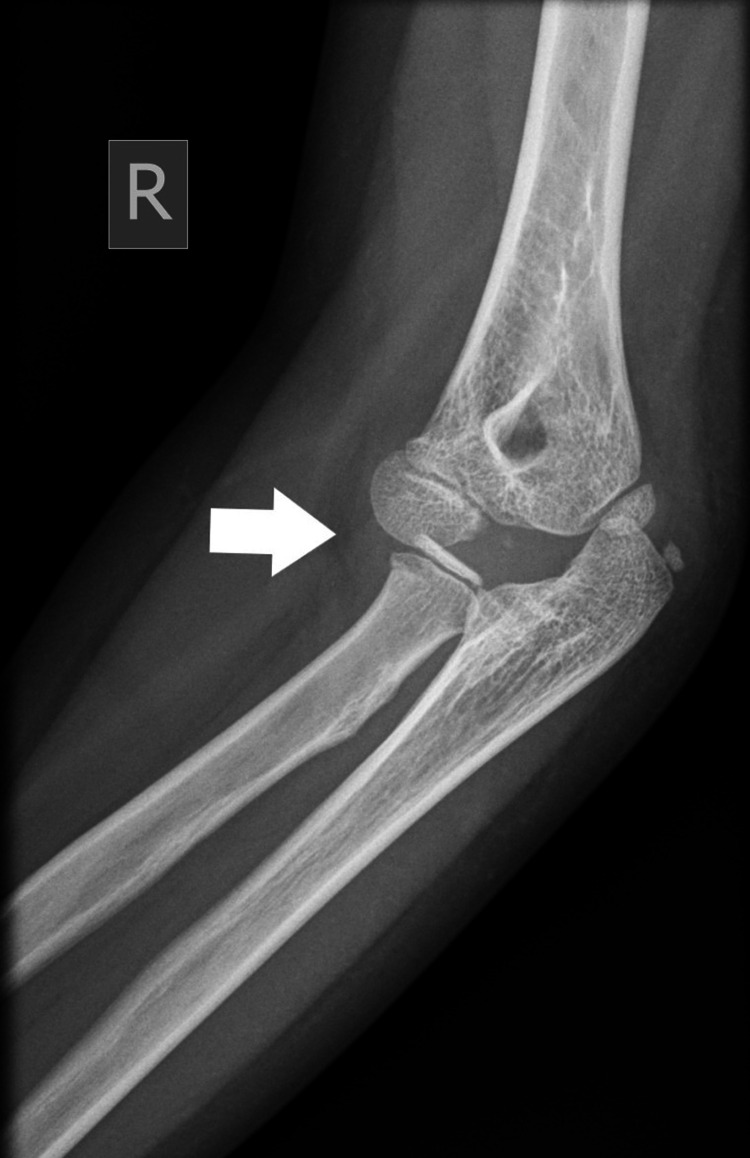
Right (R) posterior elbow dislocation (white arrow) without periarticular fracture (view 2).

**Figure 6:**
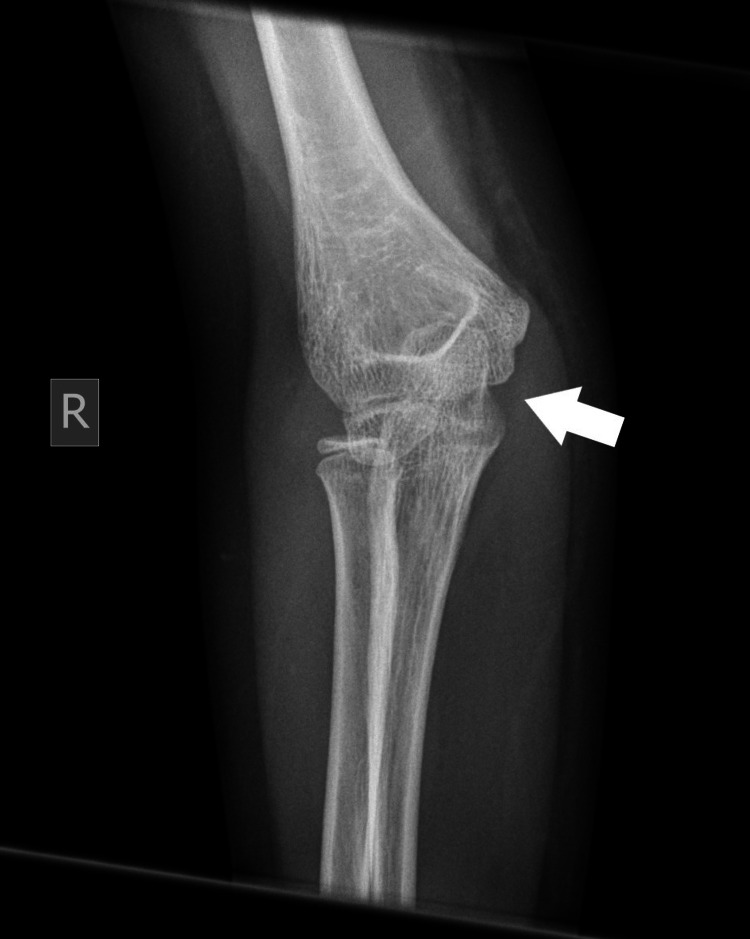
Right (R) posterior elbow dislocation (white arrow) without periarticular fracture (view 3).

Plain radiographs with standard projections (anteroposterior [AP] and lateral views) confirmed posterolateral elbow dislocation bilaterally with no signs of evident fractures. Identification of the bony structures was performed, and meticulous control and confirmation of the secondary ossification centers expected for the patient's age was done to exclude any secondary damage (Figures 1-6).

**Figure 7:**
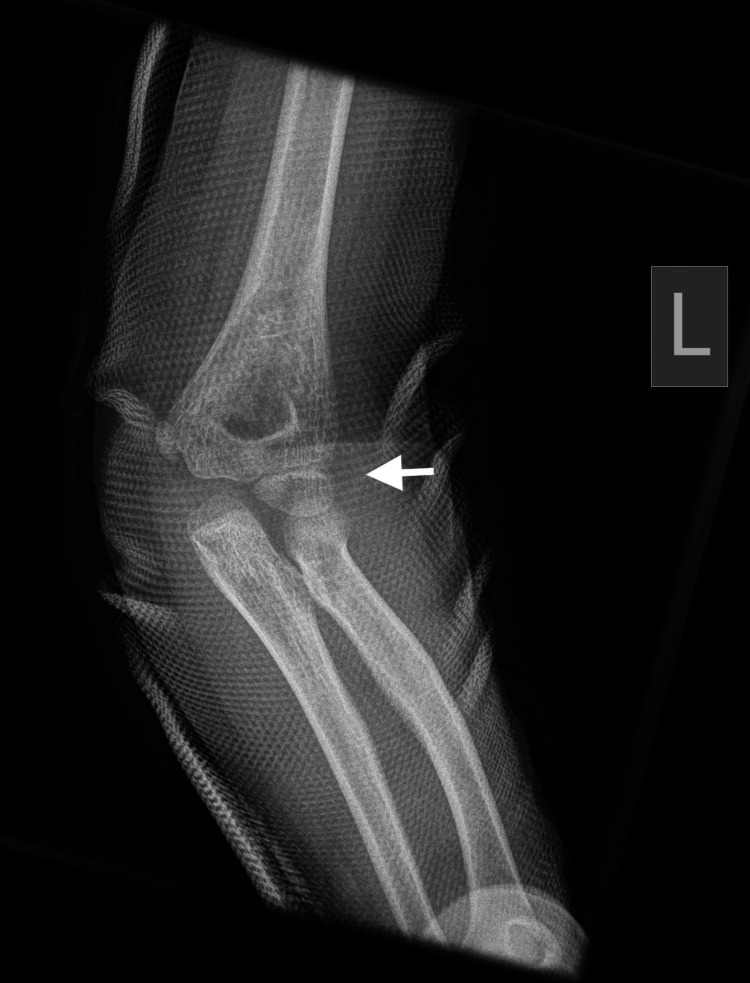
Postoperative x-ray of left (L) elbow after closed reduction (white arrow) (view 1).

**Figure 8:**
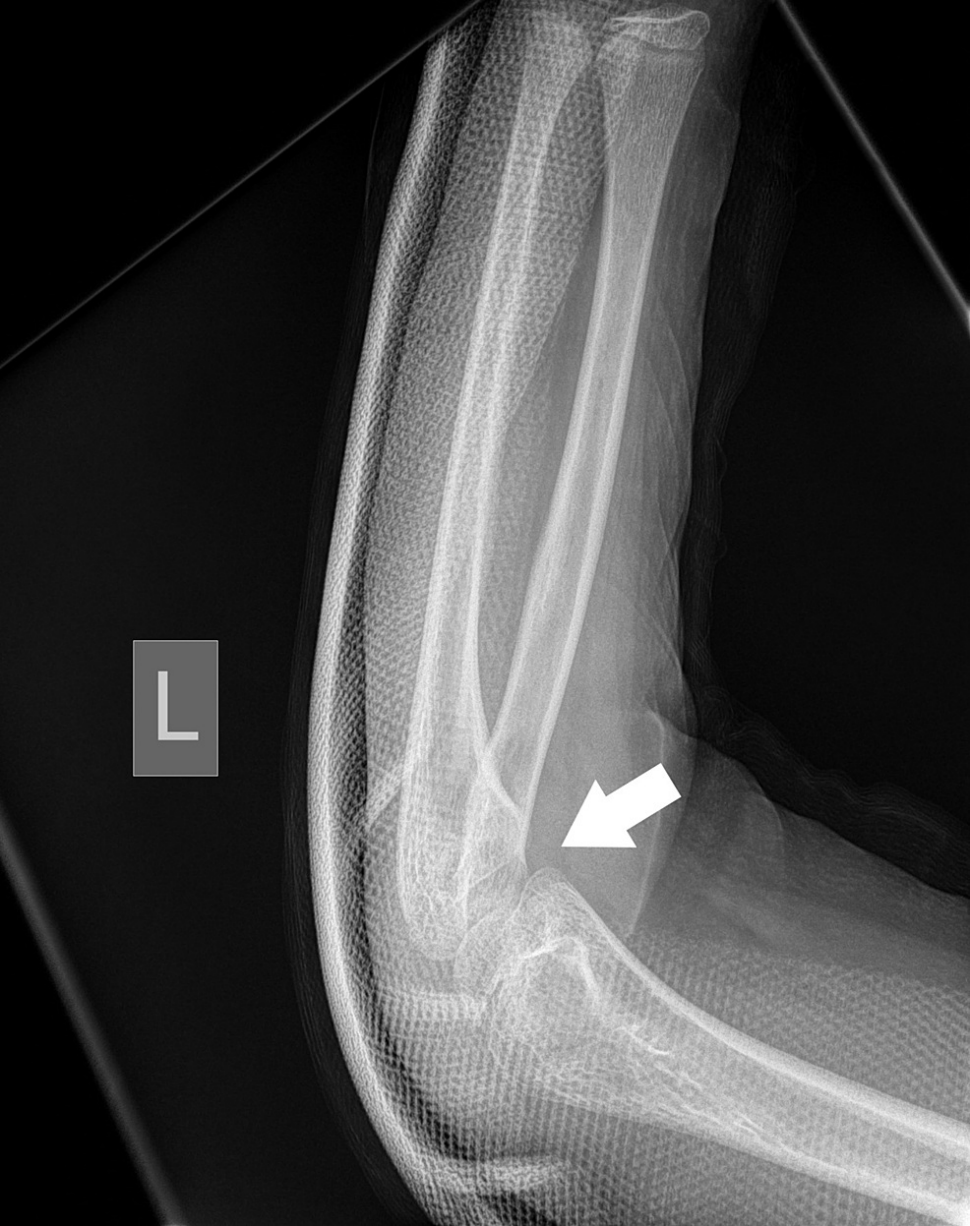
Postoperative x-ray of left (L) elbow after closed reduction (white arrow) (view 2).

**Figure 9:**
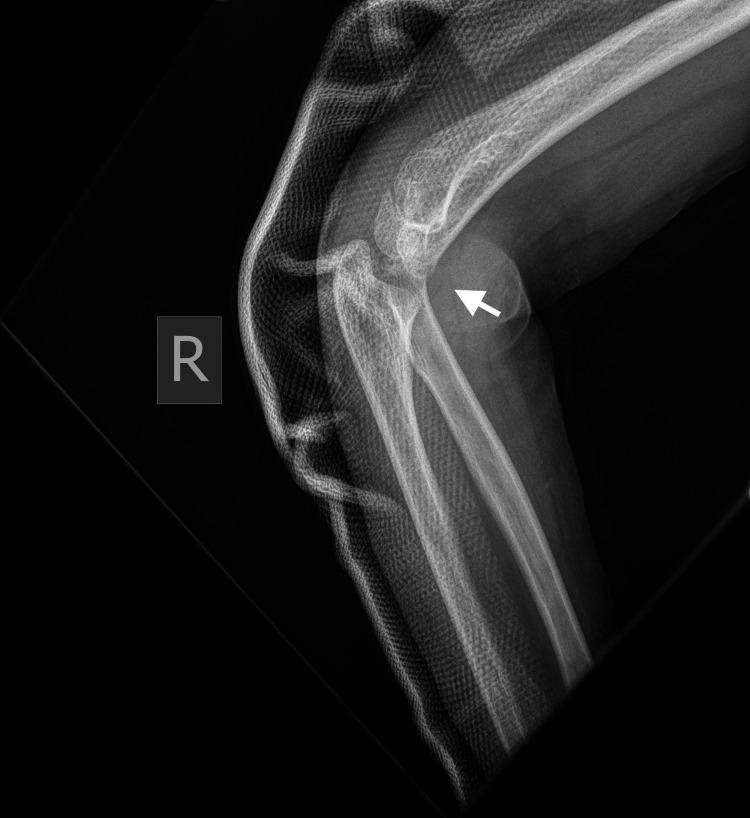
Postoperative x-ray of right (R) elbow after closed reduction (white arrow) (view 1).

**Figure 10:**
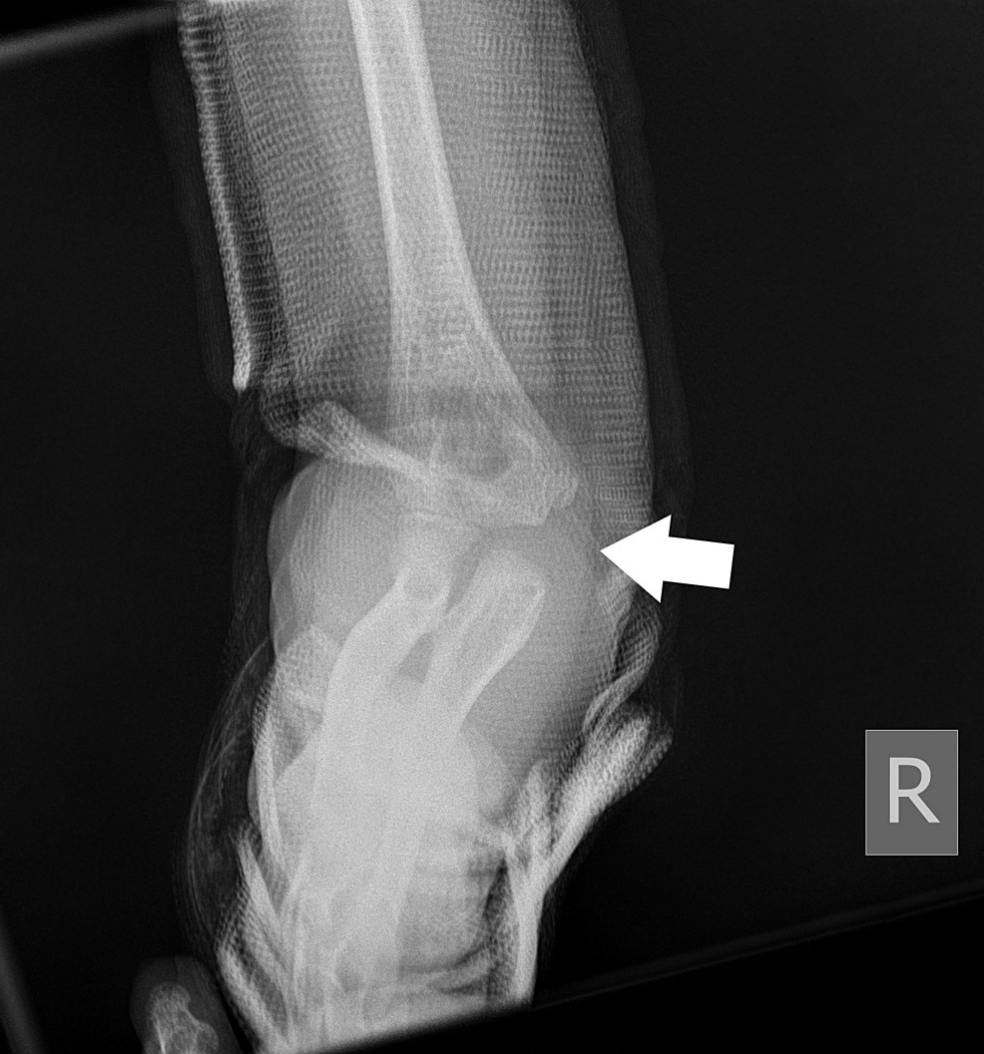
Postoperative x-ray of right (R) elbow after closed reduction (white arrow) (view 2).

In the emergency department, the upper limbs were immobilized in a provisional plaster with the elbows in a light hyperextension and neutral rotation of the forearms to reduce any movement and relieve the pain. The reduction was achieved under sedation in the operating theater and muscle relaxation with the patient in the beach chair position and with access to fluoroscopy during the whole procedure. The maneuver included gentle manipulation of the joints by slightly rotating, distracting and giving a flexion jerk to the joint. The audible and palpable "click" sign and the complete restoration of the arch of motion with the appropriate imaging confirmed the reduction as well as achievement of ligamentotaxis. Postoperatively, the patient retained both elbows in a functional position, stabilized with dorsal braces and collar and cuff to suspend the extremities (Figures 7-10).

Removal of any support took place in three weeks post-reduction, followed by mild kinesiotherapy and gradual return to moderate athletic activities. The patient achieved a full range of motion with no pain and excellent ligament stability. At the six-week follow-up, new radiographs were obtained, with normal findings.

## Discussion

Unilateral elbow dislocation is an infrequent injury accounting for only 3%-6% of all pediatric elbow injuries. The average age of pediatric patients at the time of injury is 9.9 years, with a gender ratio of 3.8, favoring females [3]. A thorough search was performed in PubMed, Ovid MEDLINE, Embase, Web of Science and Cochrane, using the search term “((bilateral[Title/Abstract]) OR simultaneous[Title/Abstract]) AND (elbow*[Title/Abstract]) AND ((dislocation*[Title/Abstract]) OR (fracture*[Title/Abstract]))”.There have only been reported 24 cases of simultaneous bilateral elbow dislocations since 1957 in the literature for all age groups (including adults, adolescents, and children). Pediatric bilateral elbow dislocations are even rarer as there have only been reported six other cases regarding patients under 18-years-old, five with concurrent periarticular fractures and one without a fracture [4-9]. The latter, which reported a 16-year-old child in 1969, had a similar mechanism of injury (falling with outstretched arms) and resembled the clinical presentation (acute pain and lack of movements due to sharp pain with x-rays featuring bilateral posterior dislocation of the elbows) with the difference of the joint being slightly more edematous and the arms to appear shortened due to the posterior displacement of the forearms [4]. No information was provided about the skeletal maturity of that patient, potential comorbidities or any signs of ligamentous laxity. Our case presented here is the first to describe simultaneous bilateral elbow dislocation without periarticular fractures in a pediatric patient under 10 years old, immature and with no clinical signs of puberty.

Bilateral elbow dislocations happen mostly in athletes, especially during gymnastics [4,7]. The usual mechanism of the fracture is falling with extended elbows, which occurred in 16 out of 24 cases. As suggested in other case reports, joint hyperlaxity may be implicated with simultaneous bilateral elbow dislocation [3]. The significantly higher degree of general joint laxity in girls of all ages than in boys may also explain the higher risk of injury in female patients. The review sample is very small, though to support the above etiopathogenesis. In our case, the patient had normal joint laxity, as mentioned before and documented with the application of the Beighton scale [2].

Bilateral elbow dislocations have also been related to hormonal changes during the menstrual period or adolescence before the first menstruation occurs [3]. There has been reported a shift in the knee joint laxity in female patients during menstruation. The child presented in our case was very young to relate the injury with the hormonal changes happening in the early pre-pubertal age.

The management of bilateral elbow dislocation is similar to the treatment of unilateral elbow dislocation. The operative treatment seems to offer no statistically different outcomes in pediatric patients than nonoperative treatment in Mayo Elbow Performance score (MEPS), Quick Disabilities of the Arm, Shoulder and Hand (QuickDASH) Sport and Music Module scores [10]. A short duration of immobilization is usually recommended to achieve the best results. Longer than three weeks of immobilization may result in worse function, mainly impacting the extension of the elbow. Periarticular ossification may occur after the immobilization but with no effect in loss of motion [11]. In our case, we performed closed reduction under sedation, and the result was excellent, as suggested by the postoperative x-rays and the restored range of motion. In the similar case of 1969 (pediatric bilateral elbow dislocation without periarticular fracture), the patient's dislocation was reduced under ether-oxygen anesthesia with similar postoperative results. In that case, the patient was hospitalized for 19 days.

The long-term outcomes of bilateral elbow dislocation cases are promising, with reports of full recovery and return to recreation and sports activities. From the six pediatric cases, the two reported full range of motion [4,7], three reported some loss of motion range [5,8,9] and one did not report any outcome [6]. The earliest return to sports activities was six weeks [9], with most patients returning in three to six months after injury.

## Conclusions

Bilateral elbow dislocation is a very scarce injury with limited reported cases in the literature especially in the pediatric population. The initial diagnostic approach should be very cautious and examine meticulously the joint as well as the neurovascular structures. Especially for young pediatric patients, the secondary ossification centers should be evaluated with scrutiny according to the respective age group. At the follow-up, estimation of the range of motion should be made as well as the muscle strength and the patient should slowly return to activities usually after kinesiotherapy sessions.

Overall, bilateral elbow dislocation is a rare injury that requires specialized care and proper treatment. We report the youngest patient with this infrequent injury, and we hope to offer some insight on dealing with this kind of injury.

## References

[1] Saeed W, Waseem M (2021). Elbow fractures overview. StatPearls [Internet].

[2] Beighton P, Solomon L, Soskolne CL (1973). Articular mobility in an African population. Ann Rheum Dis.

[3] Schubert I, Strohm PC, Zwingmann J (2019). Simple elbow dislocations in children : systematic review and meta-analysis. (Article in German). Unfallchirurg.

[4] Kovrizhnyĭ VG, Savvin EM (1969). A case of simultaneous bilateral luxation in the elbow joint. (Article in Russian). Klin Khir.

[5] Bauer S, Dunne B, Whitewood C (2012). Simultaneous bilateral elbow dislocation with bilateral medial epicondyle fractures in a 13-year-old female gymnast with hyperlaxity. BMJ Case Rep.

[6] Tayob AA, Shively RA (1980). Bilateral elbow dislocations with intra-articular displacement of the medial epicondyles. J Trauma.

[7] Jensen UH, Rud B (1983). Bilateral dislocation of the elbows. (Article in Danish). Ugeskr Laeger.

[8] Holloway NJ, Shanker H, Campbell AC (2006). Bilateral posterior elbow dislocation with heterotopic ossification in a child. Injury Extra.

[9] Sybert MW, Hennrikus WL (2017). Bilateral medial epicondyle fractures with elbow dislocations in an adolescent female athlete. Trauma.

[10] Nussberger G, Schädelin S, Mayr J, Studer D, Zimmermann P (2018). Treatment strategy and long-term functional outcome of traumatic elbow dislocation in childhood: a single centre study. J Child Orthop.

[11] Schippinger G, Seibert FJ, Steinböck J, Kucharczyk M (1999). Management of simple elbow dislocations. Does the period of immobilization affect the eventual results?. Langenbecks Arch Surg.

